# *In vitro *norepinephrine significantly activates isolated platelets from healthy volunteers and critically ill patients following severe traumatic brain injury

**DOI:** 10.1186/cc6931

**Published:** 2008-06-18

**Authors:** Christoph Tschuor, Lars M Asmis, Philipp M Lenzlinger, Martina Tanner, Luc Härter, Marius Keel, Reto Stocker, John F Stover

**Affiliations:** 1 Surgical Intensive Care Medicine, University Hospital Zuerich, Raemistrasse 100, CH 8091 Zuerich, Switzerland; 2 Institute for Clinical Hematology, University Hospital Zuerich, Raemistrasse 100, CH 8091 Zuerich, Switzerland; 3 Division of Trauma Surgery, Department of Surgery, University Hospital Zuerich, Raemistrasse 100, CH 8091 Zuerich, Switzerland

## Abstract

**Introduction:**

Norepinephrine, regularly used to increase systemic arterial blood pressure and thus improve cerebral perfusion following severe traumatic brain injury (TBI), may activate platelets. This, in turn, could promote microthrombosis formation and induce additional brain damage.

**Methods:**

The objective of this study was to investigate the influence of norepinephrine on platelets isolated from healthy volunteers and TBI patients during the first two post-traumatic weeks. A total of 18 female and 18 male healthy volunteers of different age groups were recruited, while 11 critically ill TBI patients admitted consecutively to our intensive care unit were studied. Arterial and jugular venous platelets were isolated from norepinephrine-receiving TBI patients; peripheral venous platelets were studied in healthy volunteers. Concentration-dependent functional alterations of isolated platelets were analyzed by flow cytometry, assessing changes in surface P-selectin expression and platelet-derived microparticles before and after *in vitro *stimulation with norepinephrine ranging from 10 nM to 100 μM. The thrombin receptor-activating peptide (TRAP) served as a positive control.

**Results:**

During the first week following TBI, norepinephrine-mediated stimulation of isolated platelets was significantly reduced compared with volunteers (control). In the second week, the number of P-selectin- and microparticle-positive platelets was significantly decreased by 60% compared with the first week and compared with volunteers. This, however, was associated with a significantly increased susceptibility to norepinephrine-mediated stimulation, exceeding changes observed in volunteers and TBI patients during the first week. This pronounced norepinephrine-induced responsiveness coincided with increased arterio-jugular venous difference in platelets, reflecting intracerebral adherence and signs of cerebral deterioration reflected by elevated intracranial pressure and reduced jugular venous oxygen saturation.

**Conclusion:**

Clinically infused norepinephrine might influence platelets, possibly promoting microthrombosis formation. *In vitro *stimulation revealed a concentration- and time-dependent differential level of norepinephrine-mediated platelet activation, possibly reflecting changes in receptor expression and function. Whether norepinephrine should be avoided in the second post-traumatic week and whether norepinephrine-stimulated platelets might induce additional brain damage warrant further investigations.

## Introduction

In clinical routine, norepinephrine is used to increase and maintain arterial blood pressure in predefined ranges with the aim of improving organ perfusion. Apart from its vascular smooth muscle cell α_1 _adrenergic targets mediating arteriolar vasoconstriction with subsequent increase in arterial blood pressure [1], norepinephrine may bind to α_2a _adrenergic receptors located on platelets [2]. Stimulation of α_2a _adrenergic receptors, in turn, could activate circulating platelets as reflected by surface expression of CD62P (P-selectin), conformational changes of the GPIIb/IIIa receptor, shedding of platelet-derived microparticles [3,4], and soluble adhesion molecules (sP-selectin). These alterations, in turn, are capable of activating platelets, leukocytes, and endothelial cells [5] in a self-perpetuating manner. Thus, there is an increasing risk for local microthrombosis formation, especially in the presence of injured endothelial cells with local activation of platelets, fibrin deposition, and binding of von Willebrand factor [2] with concomitant activation of immunocompetent cells [6]. Subsequently, this could promote ensuing edema progression and cell damage in pre-injured organs. In this context, severe traumatic brain injury (sTBI) is associated with endothelial damage and local microthrombosis formation which contribute to impaired cerebral microcirculation [7-9]. These microcirculatory changes may be amplified by additional norepinephrine-mediated platelet activation, adhesion, and aggregation since norepinephrine with its α_2a _adrenergic stimulation of platelets is routinely infused to elevate cerebral perfusion pressure (CPP) following sTBI. Consequently, anticipated neuroprotection by increasing CPP might be compromised due to sustained norepinephrine-induced platelet activation.

The aims of the present descriptive study were to assess whether (a) norepinephrine increases signs of functional activation in isolated platelets in a concentration-dependent manner, (b) there are differences between arterial and jugular venous platelets, (c) these alterations are time-dependent during the course of sTBI, and (d) arterio-jugular venous differences (AJVDs) are associated with signs of cerebral worsening in critically ill patients suffering from sTBI. To this end, changes in surface expression of P-selectin and intracellular prothrombotic platelet-derived microparticles of isolated platelets taken from healthy controls and sTBI patients were determined by flow cytometry.

## Materials and methods

To determine the potential stimulatory effects of norepinephrine on platelets, platelets were isolated from healthy controls and patients suffering from sTBI. Following informed written consent by the volunteers and the relatives of the sTBI patients, respectively, blood samples were drawn from 36 volunteers and 11 sTBI patients according to the protocol approved by our local ethics committee.

The study was conducted from January to October 2006 at the University Hospital of Zuerich. Patients were included if they were sedated and had received an intracranial pressure (ICP) probe and a jugular venous catheter. Continuous assessment of jugular venous oxygen saturation (SjvO_2_) as well as the intermittent analysis of arterio-jugular venous glucose and lactate differences by routine blood gas analysis were used to guide therapeutic interventions following sTBI. Patients younger than 18 and older than 65 years were not enrolled. Patients with a history of previous TBI as well as intake of drugs known to influence platelet function (for example, aspirin, ibuprofen, and clopidrogel) within 8 days before trauma were excluded. Patients with a known history of alcohol abuse, drug abuse, as well as metabolic disorders and renal/hepatic dysfunction were also excluded.

### Age- and gender-dependent influences

To rule out age- and gender-dependent influences, female and male volunteers were grouped in three age clusters: 20 to 30, 31 to 40, and 41 to 50 years, with 6 volunteers per gender and age cluster, resulting in a total of 36 volunteers.

### Physiologic data

To ensure that recruited volunteers were healthy, a carefully structured interview was conducted and various variables (for example, blood pressure, pulse, temperature, and peripheral oxygen saturation) were determined before platelets were isolated and stimulated *in vitro*. Volunteers with a recent history of fever, surgery, or intake of drugs possibly influencing platelet function (for example, aspirin and clopidrogel) were excluded.

### Blood samples

#### Volunteers

In healthy volunteers, blood was drawn once from the cubital vein with 21-gauge needles. Blood was collected in commercially available tubes containing 3.2% sodium citrate (Sarstedt, Nümbrecht, Germany). While 2 mL was used to determine differential blood count by the Institute for Clinical Hematology at the University Hospital Zuerich, 4 mL was used to investigate functional changes in isolated platelets. Approximately 0.5 mL of blood was used for venous blood gases using the Radiometer ABL 610^® ^(Radiometer A/S, Brønshøj, Denmark). Fasted volunteers were investigated between 8 and 10 a.m., following a resting period of 30 minutes upon arrival. Blood sampling as well as questioning and assessment of physiologic variables were performed by the same investigator.

#### Patients

In sTBI patients, arterial and jugular venous blood (6 mL each) was drawn using the same tubes as in the volunteers. Blood samples were drawn once daily up to 2 weeks until removal of the jugular venous catheter. Differential blood counts were performed by the Institute for Clinical Hematology at the University Hospital Zuerich once daily, while platelets were isolated and treated by a standardized protocol as outlined below. Changes in cerebral metabolism were determined by assessing alterations in glucose, lactate, and SjvO_2 _measured by routine blood gas analysis of arterial and jugular venous blood drawn at the same time point. Before the actual blood samples used for laboratory and *in vitro *analysis were drawn, the first 2 mL of blood was discarded to minimize the potential impact of local thrombus formation at the tip of the catheters which could develop over time.

### Intensive care unit treatment following severe traumatic brain injury

Following placement of an ICP probe, patients with sTBI were treated in the intensive care unit (ICU) according to a standardized protocol. Routine treatment and decision making were not influenced by the present investigations, and the obtained data were not integrated in the current treatment concept. Continuously infused midazolam (Dormicum^® ^and fentanyl (Sintenyl^® ^were tapered according to ICP values. Volume and norepinephrine administration were adjusted to maintain CPP values above 70 mm Hg. Patients did not receive heparin or low-molecular-weight heparin. All flush systems were maintained without heparin.

### Isolation of platelets

Platelet activation was measured in platelet-rich plasma (PRP) using monoclonal antibodies and three-color flow cytomtery. Within 30 minutes of blood withdrawal, samples were centrifugated at 5,000 rounds per minute for 15 minutes. Thereafter, 5 μL of PRP was added to a 12 × 75-mm tube containing 15 μL of each of the following fluorescent-labelled monoclonal antibodies: CD61-fluorescein isothiocyanate and CD62P-phycoerythrin. CD62P (P-selectin) is an antigen present on the surface of activated platelets [10]. Anti-CD61 recognizes the platelet glycoprotein receptor, GPIIIa, which is found on all resting and activated platelets and which is used to identify platelets.

After 20 minutes of incubation with monoclonal antibodies in the dark at room temperature, 1 mL of 1% paraformaldehyde was added to each tube for fixation of platelets. Mouse immunoglobulin G 1 (fluorescein isothiocyanate) and phycoerythrin were used as isotype controls. Antibodies and isotype controls were purchased from Becton Dickinson Immunocytometry Systems (San Jose, CA, USA). All samples were analyzed within 90 minutes on a FACSscan flow cytometer (Becton Dickinson, Mountain View, CA, USA) using Cell Quest^® ^software (Becton Dickinson Immunocytometry Systems). Flow cytometer performance used to analyze microparticles was verified employing 1-μm calibration beads (Bangs Laboratories, Inc., Fishers, IN, USA).

A total of 5,000 CD61-positive events were collected with all light scatter and fluorescence parameters in a logarithmic mode. Platelets were gated on the basis of light scatter and CD61 expression. Activated platelets were defined as the percentage of CD61-positive events expressing the activated confirmation of P-selectin (CD62P). Platelet-derived microparticles were also measured and identified as CD61-positive events in a gate obtained using uniform microspheres of 7.4 μm in diameter (Bangs Laboratories, Inc.).

### Stimulation of isolated platelets

Double samples of isolated peripheral venous, jugular venous, and arterial platelets were incubated for 20 minutes with different norepinephrine concentrations (Noradrenaline Sintetica 0.1%; Sintetica S.A., Mendrisio, Switzerland) ranging from 10 nM to 100 μM. The same norepinephrine as employed in the routine treatment in our ICU was used for the *in vitro *stimulation. Thrombin receptor-activating peptide (TRAP) (Becton Dickinson Immunocytometry Systems), known to maximally activate platelets, served as a positive control. Upon stimulation, changes in expression of P-selectin-positive platelets and changes in the number of CD61-positive platelet-derived microparticles were assessed to reveal the degree of platelet activation. All samples were analyzed within 90 minutes after blood withdrawal.

### Analysis of differential blood counts

Differential blood counts were analyzed in the ISO-IEC 17025 accredited university hospital laboratory at the University Hospital Zuerich.

### Analysis of sP-selectin

sP-selectin was measured in plasma using a DuoSet^® ^ELISA [enzyme-linked immunosorbent assay] Development System (R&D Systems, Inc., Minneapolis, MN, USA) in accordance with the instructions of the manufacturer.

### Assessment of mean arterial blood pressure, intracranial pressure, cerebral perfusion pressure, arterio-jugular venous differences, drug dosage, and hydroxyethyl starch

Continuously recorded ICP, CPP, temperature, and SjvO_2 _were assessed in 1-hour intervals. Drug dosage was also determined in 1-hour intervals. A daily median was calculated using these 24 values. Daily administration of hydroxyethyl starch (HES) (Voluven^® ^was recorded. AJVDs in glucose and lactate were assessed in 4- to 6-hour intervals, allowing us to calculate a daily median. AJVDs in platelets, leukocytes, and sP-selectin were measured once daily.

### Calculation of arterio-jugular venous differences

Jugular venous values were substracted from arterial values, thus yielding the calculated AJVDs. Positive AJVDs reflect cerebral retention or uptake as the arterial levels exceed the jugular venous concentration. Negative AJVD values reveal sustained release or decreased uptake/binding within the cerebral compartment as jugular venous levels exceed arterial concentrations.

### Statistical analysis

Results are presented as median or mean ± standard error of the mean, where applicable. Differences between groups, time points, and norepinephrine concentrations were rated significant at a probability level of less than 0.05 using analysis of variance on ranks with *post hoc *multiple pairwise comparisons. Statistical analysis was performed using SigmaStat^® ^3.5 (SPSS Inc. Headquarters, Chicago, Illinois, USA). Figures were created with SigmaPlot^® ^10.0 (SPSS Inc. Headquarters, Chicago, Illinois, USA).

## Results

### Healthy controls

#### Physiologic and laboratory values

Physiologic data and laboratory values revealing that all 36 volunteers were healthy are presented in Table 1. Since there were no age- or gender-related differences (data not shown), data of all volunteers were pooled.

**Table 1:** Physiologic and laboratory data of 36 healthy volunteers

Parameters (normal values)	Median ± SEM	Range
Physiologic data		
Body mass index, kg/m^2^	24 ± 0.5	17.3–34.4
Temperature, °C	36.8 ± 0.1	35.6–37.2
SpO_2_, percentage	98 ± 0.2	95–100
Heart rate, beats per minute	80 ± 2	56–101
MABP, mm Hg	99 ± 2	78–131
HCO_3_^-^, mM	26.7 ± 0.3	21.4–28.5
Glucose, mM	5.9 ± 0.13	4.1–8.2
Lactate, mM	1.3 ± 0.08	0.7–2.4
Differential blood count		
Hemoglobin, g/dL (13.4–17.0)	14.1 ± 0.3	11.5–16.3
Platelets, 10^3^/μL (143–400)	261 ± 12	190–411
Leukocytes, 10^3^/μL (3.0–9.6)	5.9 ± 0.35	2.94–10.77
		
sP-selectin, ng/mL	63 ± 10	45–96

Due to absent differences, data from different age groups and gender were pooled. MABP, mean arterial blood pressure; SEM, standard error of the mean; SpO_2_, peripheral oxygen saturation.

#### *In vitro *norepinephrine stimulation of isolated platelets

**Figure 1:**
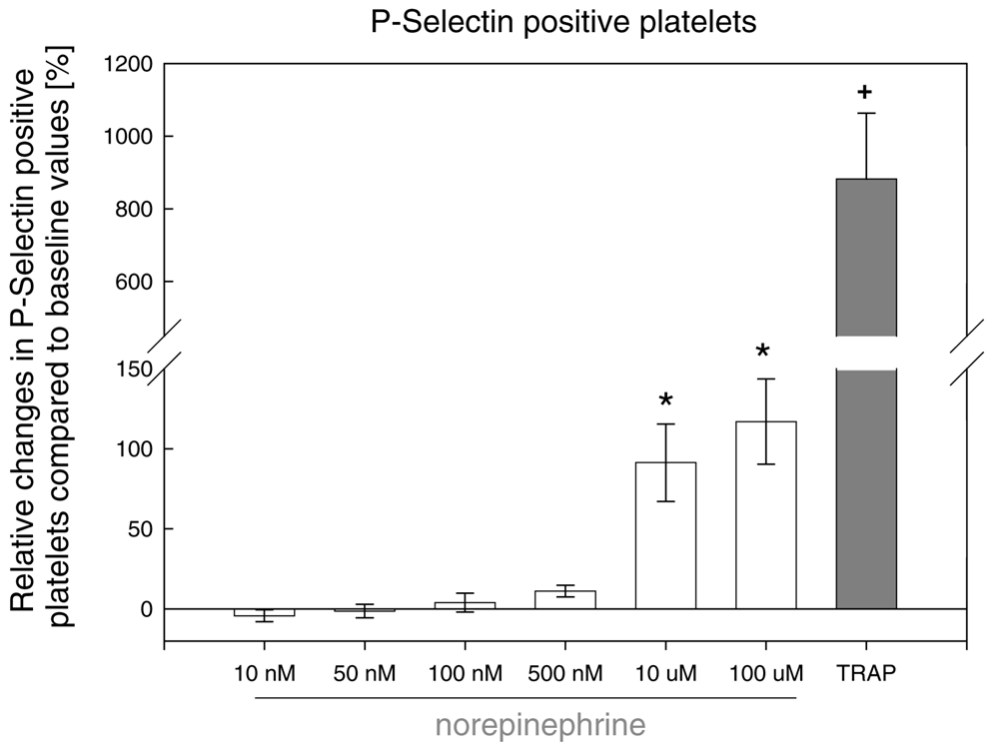
Effect of norepinephrine and thrombin receptor-activating peptide (TRAP) on surface expression of P-selectin in platelets isolated from healthy controls. Norepinephrine, in a concentration-dependent manner, increased the number of P-selectin-positive platelets, which was significant only at norepinephrine concentrations of greater than or equal to 10 μM. Maximal increase was induced with TRAP. ^+^*P *<0.001 TRAP versus norepinephrine; * *P *<0.001 norepinephrine of 10 and 100 μM versus norepinephrine of less than 10 μM; analysis of variance on ranks.

**Figure 2:**
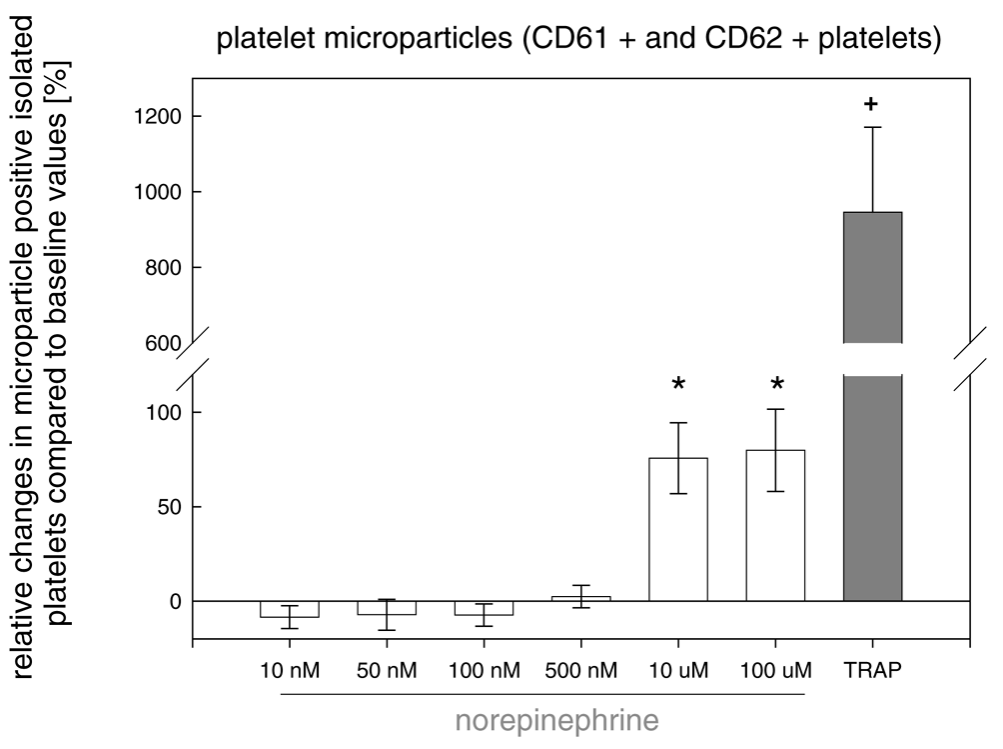
Significant concentration-dependent influence of norepinephrine and thrombin receptor-activating peptide (TRAP) on platelet microparticles isolated from healthy controls. This effect was significant only at norepinephrine concentrations of greater than or equal to 10 μM with a maximal increase induced with TRAP. ^+^*P *<0.001 TRAP versus norepinephrine; * *P *<0.001 norepinephrine of 10 and 100 μM versus norepinephrine of less than 10 μM; analysis of variance on ranks.

*In vitro *stimulation of isolated platelets with norepinephrine showed a significant concentration-dependent increase in P-selectin-positive (Figure 1) and microparticle-positive (Figure 2) platelets compared with isolated platelets which were not stimulated by norepinephrine under baseline conditions. Incubation with TRAP significantly and maximally increased P-selectin and microparticle expression compared with baseline values of unstimulated platelets (Figures 1 and 2). Overall, there were no age- or gender-dependent differences (data not shown).

### Patients with severe traumatic brain injury

Demographic data of the investigated critically ill patients suffering from sTBI are presented in Table 2. Changes in absolute blood platelet and leukocyte counts, AJVDs of platelets, leukocytes, glucose, and lactate as well as mean arterial blood pressure, ICP, CPP, SjvO_2_, temperature, and average drug dosage are presented in Table 3. Data were pooled for the first and second week. During the second week, absolute platelet and leukocyte counts were significantly increased. Whereas platelets remained within normal limits, leukocytes surpassed the upper limit of normal values. Whereas ICP was significantly increased, CPP, SjvO_2_, and temperature were significantly decreased during the second week compared with the first week. These changes, however, remained within clinically acceptable limits. Administered drug dosages were similar for norepinephrine, midazolam, and fentanyl during the first and second week. In a total of 751 SjvO_2_, CPP, and ICP values which were recorded at the same time as jugular venous blood gas analysis only 0.4% SjvO_2 _were less than 50%, 0.1% of CPP values were less than 60 mm Hg, and 17% of ICP was greater than 20 mm Hg. In eight of the 11 patients, pneumonia was diagnosed on (a median of) 8.5 days after trauma (range 3 to 13 days). In 1 patient (#3), bacteremia with coagulase-negative *Staphylococcus aureus *was diagnosed. In 1 multiply injured patient (#8), pulmonary embolism was diagnosed clinically and verified radiologically on day 12 after trauma after the patient was mobilized. A deep venous thrombosis was not found. A vena cava filter was inserted and removed after 14 days. Thereafter, the patient had an uneventful recovery.

**Table 2:** Demographic data of 11 consecutively investigated critically ill patients suffering from severe traumatic brain injury

Patient	Age, years	Gender	Initial GCS	Brain lesions	Additional injuries	AIS head	ISS total	Length JB, days	ICU stay, days	eGOS
1	23	Female	15	Mixed	Thorax, skin	5	45	16	41	8
2	54	Male	3	Mixed	-	5	25	5	16	7
3	32	Male	3	Mixed	Thorax, extremities	5	57	24	51	7
4	41	Female	6	Mixed	-	5	25	18	26	8
5	64	Male	6	Mixed	Thorax, abdomen	5	45	7	10	1
6	53	Male	14	Multiple contusions	-	5	25	2	3	1
7	19	Male	12	Mixed	-	5	25	20	27	7
8	49	Male	15	Isolated EDH	Thorax, spine, extremities	5	38	7	21	5
9	51	Male	15	Isolated EDH	Thorax, spine, extremities, pelvis, skin	4	41	4	16	5
10	41	Male	10	Mixed	Thorax, spine, extremities	5	38	10	17	6
11	43	Male	14	Isolated contusion	Face, skin, extremities	5	33	6	12	7
Median, range	43, 23–64	2 females/9 males	11, 3–15	7 mixed lesions	7 with additional injuries	5, 4–5	38, 25–57	7, 2–24	17, 3–51	7, 1–8

Due to individual clinical courses, the jugular venous catheter was removed at different days, resulting in a lower number of patients during the second week (n = 5 versus n = 11, first week). AIS, abbreviated injury score; EDH, epidural hematoma; eGOS, extended Glasgow Outcome Score; GCS, Glasgow Coma Scale score determined at the site of accident; ICU, intensive care unit; ISS, injury severity score; JB, jugular bulb.

**Table 3:** Changes in laboratory and clinical variables following severe traumatic brain injury

	First week	Second week	*P *value
Laboratory values			

Platelets, × 10^3^/μL	150 ± 6	215 ± 10^a^	<0.001
Lowest values	128 ± 14; day 1		
Highest values		224 ± 23; day 14	
Leukocytes, × 10^3^/μL	8.7 ± 0.4	12.3 ± 1^a^	<0.01
C-reactive protein, mg/L	121 ± 26	133 ± 23	NS
Interleukin-6, ng/L	142 ± 40	78 ± 21	NS

Calculated arterio-jugular venous differences			

AJVD platelets, × 10^3^/μL	1.5 ± 0.9	5.8 ± 2^a^	<0.01
AJVD leukocytes, × 10^3^/μL	-0.12±0.05	-0.02±0.1^a^	<0.03
AJVD glucose, mM	0.33 ± 0.02	0.43 ± 0.04^a^	<0.04
AJVD lactate, mM	-0.03±0.006	-0.06±0.01^a^	<0.04
AJVD sP-selectin, pg/mL	454 ± 932	700 ± 1,254	NS

Neuromonitoring			

Mean arterial pressure, mm Hg	97 ± 1	96 ± 1	NS
Intracranial pressure, mm Hg	13 ± 0.7	16 ± 0.5^a^	0.019
Cerebral perfusion pressure, mm Hg	83 ± 1	80 ± 1	NS
SjvO_2_, percentage	76 ± 1	69 ± 1^a^	<0.001
Temperature, °C	36.2 ± 0.1	35.5 ± 0.1^a^	<0.001

Pharmacologic treatment/platelet transfusions			

Norepinephrine, μg/minute	7 ± 0.64	7.2 ± 1.03	NS
Fentanyl, mg/hour	0.6 ± 0.05	0.59 ± 0.08	NS
Midazolam, mg/hour	62 ± 5	59 ± 8	NS
Platelet transfusions, ml	300 ± 227 (n = 4)	0	
HES 130/0.4, mL (Voluven^®^			
Cumulative	11,935 ± 1,826^a^	3,000 ± 2,100	<0.001
Daily average	1,571 ± 260^a^	429 ± 300	<0.001

Positive arterio-jugular venous differences (AJVDs) reflect cerebral uptake, while negative AJVD values unmask release or decreased uptake/binding. Values are expressed as mean ± standard error of the mean. ^a^Differences are rated significant at the corresponding levels of significance using the *t *test or Mann-Whitney test, respectively. a, significant differences; HES, hydroxyethyl starch; NS, not significant; SjvO_2_, jugular venous oxygen saturation.

#### Arterio-jugular venous differences

AJVDs for platelets showed predominantly positive values, which increased significantly over time, exceeding the positive values calculated during the first week. AJVD values for leukocytes were predominantly negative and were significantly decreased during the second week. The positive values for AJVD in glucose showed a significant increase over time, whereas the negative values for AJVD in lactate continued to decrease during the second week. Contrary to the significant findings in absolute platelet counts and AJVD in platelets, the AJVD for sP-selectin remained unchanged despite a trend toward higher values.

#### *In vivo *measurements of isolated platelets

**Figure 3:**
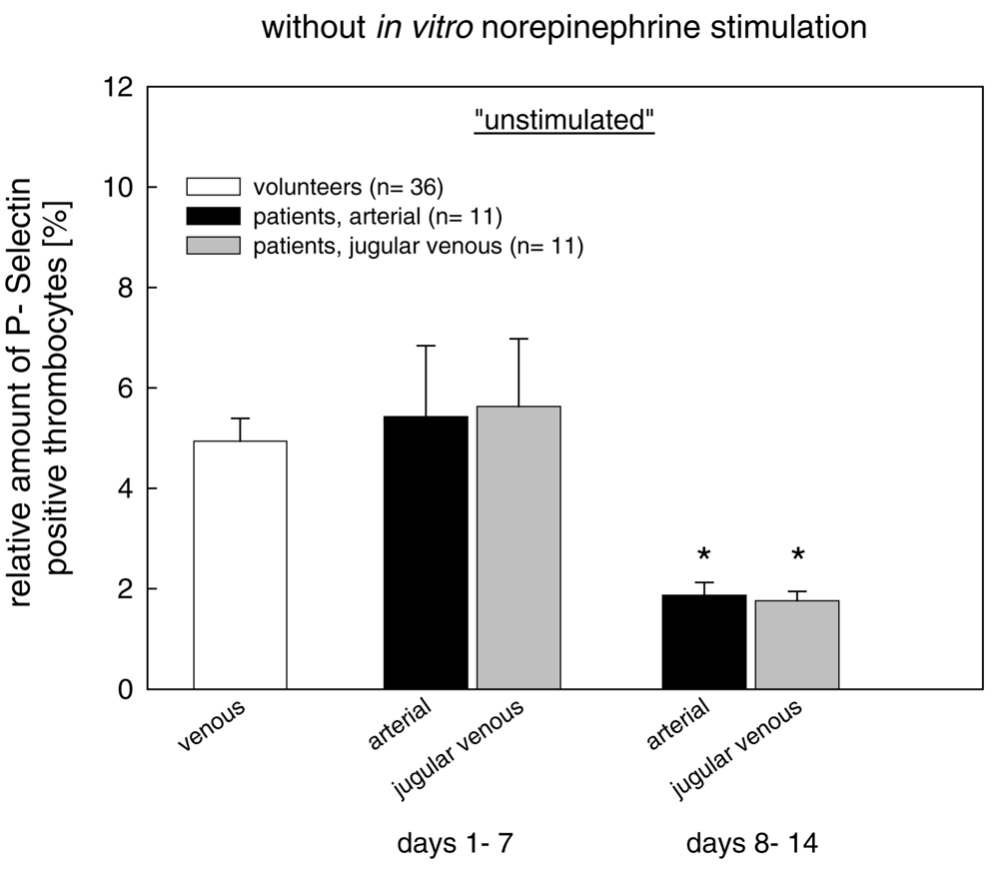
Changes in expression of surface P-selectin in platelets isolated from severe traumatic brain injury patients compared with healthy controls. The relative number of P-selectin-positive arterial and jugular venous platelets was significantly decreased during the second week. * *P *<0.05 versus controls and first week; analysis of variance on ranks.

**Figure 4:**
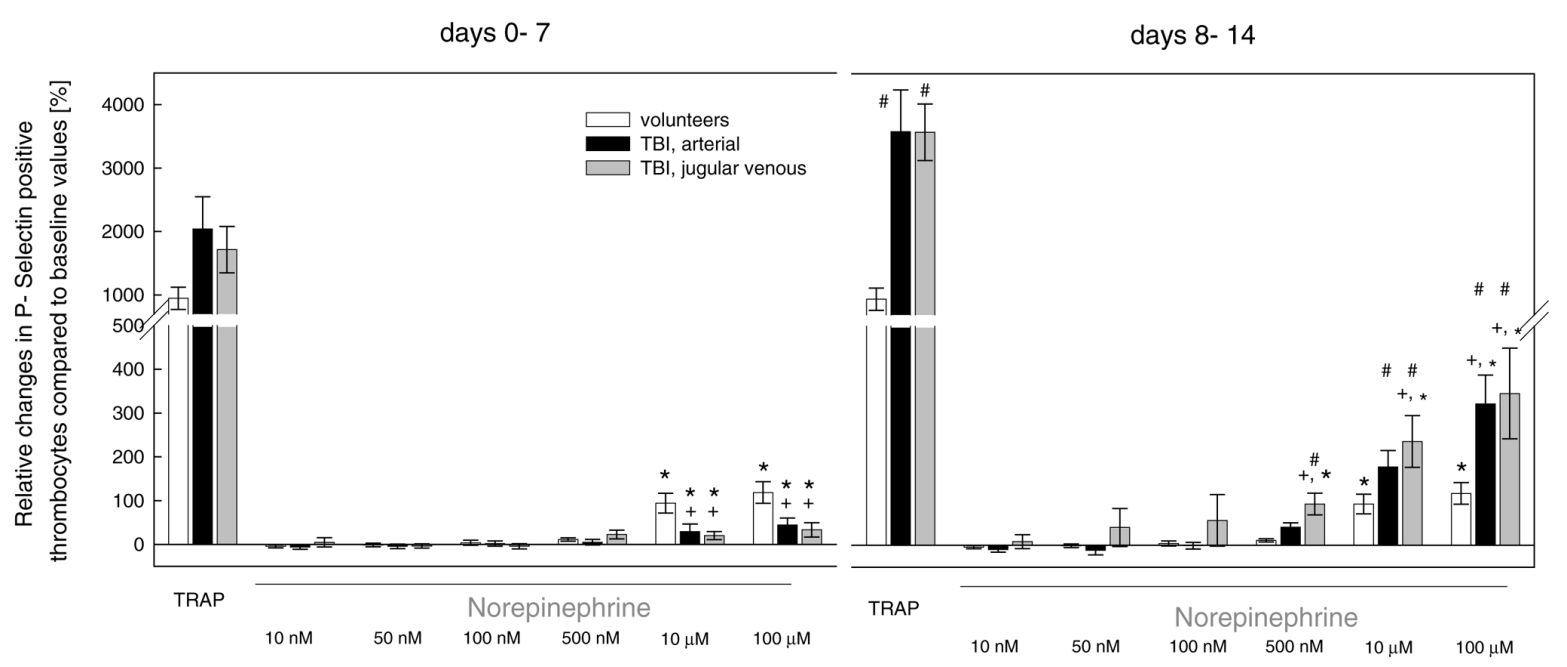
Relative increases in norepinephrine-induced expression of P-selectin in arterial (black bars) and jugular venous (grey bars) platelets isolated from severe traumatic brain injury (TBI) patients and peripheral venous platelets taken from healthy controls (white bars) expressed as a percentage of baseline values. Baseline values were determined in platelets not stimulated *in vitro *with norepinephrine. During the first week, the norepinephrine-mediated increase in P-selectin-positive platelets was significantly reduced compared with controls. In the second week, the norepinephrine-mediated increase in P-selectin expression significantly exceeded changes seen in the first week and in healthy volunteers. Overall, there was no significant difference between arterial and jugular venous platelets. During the second week, the TRAP-mediated increase in P-selectin-positive platelets significantly exceeded the TRAP-induced activation observed during the first week. ^#^*P *<0.001 second week versus first week; ^+^*P *<0.01 patients versus controls; * *P *<0.01 norepinephrine of greater than 500 nM versus norepinephrine of less than 10 μM. TRAP, thrombin receptor-activating peptide.

During the second post-traumatic week, the number of P-selectin-positive cells expressed as the relative amount of all gated platelets was significantly reduced compared with healthy controls and the first week (Figure 3). Similar changes were also observed for CD61-positive microparticles (data not shown). Incubation with TRAP, however, maximally increased the relative amount of P-selectin-positive (Figure 4) and microparticle-positive (data not shown) platelets, which was mostly sustained in platelets isolated during the second week (Figure 4). Overall, there was no significant difference between arterial and jugular venous platelets (Figures 3 and 4).

#### *In vitro *norepinephrine stimulation of isolated platelets

Upon incubation with norepinephrine, the expression of P-selectin-positive (Figure 4) and microparticle-positive (data not shown) platelets was significantly increased in a concentration-dependent manner compared with baseline values of freshly isolated platelets which were not stimulated. During the first week, however, this response was significantly attenuated compared with healthy controls. During the second week, norepinephrine-mediated increase in P-selectin-positive and microparticle-positive platelets significantly exceeded the changes observed during the first week and the corresponding alterations found in volunteers. Overall, there was a trend toward sustained stimulation in jugular venous compared with arterial platelets (Figure 4) which, however, did not reach statistical significance, due to the low number of patients (n = 5).

## Discussion

Under *in vitro *conditions, incubating isolated platelets with norepinephrine significantly and concentration-dependently increased the expression of surface P-selectin and intracellular prothrombotic microparticles, reflecting increased platelet activation. Interestingly, this response revealed a differentiated temporal profile in critically ill sTBI patients with a significantly reduced stimulation during the first week, followed by a sustained stimulatory effect during the second week. This coincided with a marked increase in circulating platelet count and in cerebral platelet retention reflected by positive AJVD values. This, however, was not associated with an increase in jugular venous sP-selectin concentrations. Despite a trend, there was no significant difference in the norepinephrine-mediated stimulation between arterial and jugular venous platelets. In addition, signs of cerebral deterioration (that is, elevated ICP, decreased SjvO_2_, and increased cerebral lactate production) coincided with the sustained norepinephrine-mediated platelet activation in the second post-traumatic week.

### Sampling and isolation procedure

Arterial and jugular venous catheters remain in place until these catheters can or need to be removed. Over time, local thrombus formation at the tip of the catheter is possible. New daily insertions of catheters to avoid any local thrombus formation, however, are not feasible under clinical conditions due to hemodynamic instability, generalized edema formation related to capillary leakage, and a limited number of accessible vessels. Local thrombus formation at the tip of the catheters activates platelets, possibly resulting in false-positive results. As a standardized procedure to reduce the risk of possible thrombus-related confounding influences, 2 mL of blood was withdrawn and discarded before the actual blood sample was taken. Nevertheless, local activation might have occurred, possibly explaining the reduced number of P-selectin-expressing platelets during the second week. In addition to local catheter-related effects, the underlying tissue damage might have contributed to platelet activation with subsequent P-selectin shedding and sustained sP-selectin concentrations. Due to the fact that the post-traumatic significantly increased sP-selectin levels exceeded normal values by several fold, any additional shedding might remain obscured. In addition, isolation procedures can activate cells. As to our own preliminary experiments, the chosen isolation procedure is associated with an activation of less than 2%.

### Changes in platelet function following trauma

As shown by Scherer and Spangenberg [11], Jacoby and colleagues [12], and Nekludov and colleagues [13,14], plasmatic coagulation, platelet count, and platelet function are significantly and reversibly altered during the early phase following sTBI. In this context, activation of the coagulation cascade which occurs within the first hours after trauma within the injured brain [11,13] as reflected by an elevated transcranial gradient precedes systemic hypercoagulability which is followed by fibrinolytic activity. These alterations, in turn, could explain the observed decrease in platelet count and fibrinogen level and subsequent increase in thrombin-antithrombin III complex, prothrombin fragment F1+2, and D-dimer concentrations [11]. Following TBI, platelets were significantly activated in the face of depressed function as reflected by prolonged collagen/epinephrine closure times during the first 3 post-traumatic days [12]. In addition, prolonged disturbance in platelet function was significantly sustained in non-surviving patients, which underlines the pathophysiologic importance of disturbed coagulation. In conjunction with a prolonged bleeding time, platelets showed a decreased responsiveness to arachidonic acid as determined by thromboelastography [14]. As shown by the present study, functional depression in isolated platelets is expanded to 7 days following sTBI and reflects prolonged functional disturbance in thrombocytic coagulation. Clinically, however, there were no signs of coagulation disorder. Following the initial functional depression, platelet function was significantly increased in the second week following sTBI, which coincided with sustained cerebral retention of platelets and signs of disturbed cerebral perfusion. Thus, these changes clearly unmask temporally differentiated changes in platelet function which are of pathophysiologic importance.

### Functional changes in platelets over time

Under physiologic conditions, quantitative and qualitative features of platelets are tightly controlled by various mediators within the bone marrow, blood, and along the endothelial cells [15]. Following injury, excessive loss and consumption of platelets exceeding production and release from bone marrow result in a significant decrease in circulating platelets, reaching its nadir by the second post-traumatic day. Subsequent significant increase reflects upregulated compensatory production within the bone marrow aimed at normalizing the amount of circulating platelets. In this context, thrombopoietin is of crucial importance [16]. Thrombopoietin also contributes to enhanced platelet activation under clinical conditions [17]. Newly produced and freshly released platelets might be activated more easily than senescent platelets. This, in turn, might explain the preserved and exaggerated *in vitro *norepinephrine-mediated stimulation during the second week as observed in the present study. The preserved functionality in platelets despite decreased baseline P-selectin expression as found in the second week is in line with results from Michelson and colleagues [18], who showed that circulating platelets remain active for at least 24 hours following shedding of surface P-selectin. In this context, we suggest that reduced P-selectin-positive platelets in the face of signs of cerebral worsening reflect functional disturbance of the isolated platelets, assuming that platelets contribute to pathophysiologic cascades within the injured brain in these patients. While P-selectin expression determines size and stability of platelet aggregates [19], reduced surface P-selectin expression does not imply functional impairment [18]. Shedding of P-selectin reflects previous platelet activation and could result in facilitated release of various toxic mediators [20,21] which have been shown to induce and promote tissue damage. This warrants further investigations.

### Norepinephrine-mediated activation of platelets

Activation of α_2 _adrenergic receptors by norepinephrine routinely infused to elevate CPP following sTBI enhanced platelet aggregability concentration dependently and increased platelet secretion of beta-thromboglobulin during high-dose infusion [22]. In addition, norepinephrine stimulated the expression of surface P-selectin and intracellular prothrombotic microparticles. Stimulation of different surface receptors results in a stereotypic amplified activation of intracellular G-protein-mediated cascades involving the Rho/Rho-kinase pathway, phospholipase C, and protein kinase C, which are essential for conformational changes in platelet shape as well as aggregation and degranulation [23].

Despite the tedious analysis and difficult interpretation of concentrations of blood norepinephrine (due to its short half-life and fast response to changes in infusion parameters), Johnston and colleagues [24] determined the pharmacokinetic profile of norepinephrine in eight patients suffering from sTBI. Based on their findings, plasma norepinephrine levels significantly correlated with the rate of norepinephrine infusion during steady-state conditions of the norepinephrine infusion period. The average norepinephrine dose infused in the presently investigated patients ranged from 0.1 ± 0.07 to 0.16 ± 0.11 μg/kg per minute. Assuming a similar norepinephrine distribution volume and clearance in our patients, we are to expect plasma levels of between 22.98 ± 16.98 and 37.08 ± 20.15 nM/L according to the results published by Johnston and colleagues [24].

Based on the assumptions that norepinephrine exhibits minimal regional and temporal fluctuations during steady-state conditions and that *in vitro *concentrations are equally potent as those *in vivo*, it appears as if extremely high norepinpehrine doses were required to activate isolated platelets. The lowest norepinephrine concentration associated with a significant effect in the presently isolated platelets was 500 nM, which exceeded the extrapolated blood levels of 25 nM by 20-fold. Thus, it remains unclear to what extent the observed effects are also valid under *in vivo *conditions.

The fact that isolated platelets exhibited a temporally differentiated response to the same norepinephrine concentration in the first versus second week coinciding with a preserved and even increased TRAP-mediated platelet activation suggests altered susceptibility of platelet receptors. In this context, functional adaptation of platelet α_2 _adrenergic receptors in terms of receptor downregulation or upregulation might be of pharmacologic and pathophysiologic importance. Clinical as well as experimental studies have shown that elevated catecholamine concentrations are associated with a reduction in expression and affinity of α_2 _adrenergic receptors [25-28]. This also resulted in a decreased platelet aggregation response to epinephrine [29]. Intracellular adaptive processes in conjunction with regained sensitization of previously desensitized α_2 _adrenergic receptors might lead to the observed sustained *in vitro *stimulation during the second week during continuous norepinephrine stimulation following the depressed stimulation during the first week. This could also account for the stimulatory effect at a lower norepinephrine concentration compared with healthy controls (500 nM versus 10 μM).

### Influence of sedation

Sedative agents (for example, midazolam) might have contributed to the decreased expression of platelet surface P-selectin as shown by Tsai and colleagues [30] and Gries and colleagues [31]. The inhibitory mechanism of midazolam is best explained by concentration-dependent blocking of platelet aggregation, inhibition of phosphoinositide breakdown and intracellular Ca^+2 ^mobilization, increased formation of cyclic AMP, inhibition of increases in intracellular pH, and attenuated protein kinase C activation [32]. Adaptive intracellular processes upon initial midazolam-induced functional depression might have contributed to the sustained norepinephrine-mediated stimulation of platelets isolated during the second week despite the administration of amounts comparable to those in the first week.

### Influence of inflammation

Whether inflammation-induced cytokine release might have contributed to the sustained *in vitro *stimulation of isolated platelets appears doubtful since interleukin (IL)-6 levels were not significantly increased during the second week in the presently investigated patients despite significant leukocytosis. This is in line with findings reported by Leytin and colleagues [33] showing that the pro-inflammatory cytokines IL-1β, IL-6, and IL-8 did not stimulate platelets and failed to promote thrombin-mediated platelet activation. Other mechanisms related to bacterial infections, however, have been shown to activate platelets, a circumstance that was not reflected by an increase in leukocytes [34]. In those 8 patients with pneumonia and the single patient with bacteremia, there was no significant difference in baseline P-selectin expression and susceptibility to norepinephrine-mediated stimulation of isolated platelets compared with the remaining 5 patients. An inflammation-induced influence, however, needs to be specifically addressed in a larger study population.

### Influence of hydroxyethyl starch solutions

In clinical routine, colloids (for example, HES) are combined with cristalloids to maintain adequate organ perfusion and to reduce catecholamine dose by inducing normovolemia. As reported by Chen and colleagues [35], HES 130/0.4 (Voluven^®^, which is routinely used in our ICU, induced transient reduction in platelet-mediated coagulation reflected by decreased platelet membrane glycoprotein and P-selectin expression in patients undergoing elective minor surgery.

Under *in vitro *conditions, HES 130/0.4 did not influence the expression of various membrane proteins on platelets isolated from healthy volunteers [36]. Thus, decreased baseline P-selectin expression observed in the second week does not appear to be induced by HES since patients required significantly less HES 130/0.4 compared with the first week. In fact, baseline P-selectin and microparticle expression were comparable to healthy volunteers during the first week despite a significantly larger amount of HES 130/0.4 administered per day compared with the single administration of HES 130/0.4 during minor surgery as studied by Chen and colleagues [35].

### Microthrombosis, platelet activation, and secondary brain injury

Following TBI, impaired pericontusional microcirculation shows a dynamic temporal and heterogeneous regional profile with impaired as well as increased cerebral perfusion [37,38]. Impaired perfusion is related to vasoconstriction and endovascular occlusion due to microthrombosis evolving within the first 24 hours and promoting edema formation. Under experimental conditions, thrombotic occlusion is followed by spontaneous resolution during the second post-traumatic day as evidenced by histology, intravital microscopy, and laser Doppler flowmetry [7-9,39,40].

Sustained platelet adhesion and activation are functionally interwoven with activated leukocytes, thereby facilitating thrombus formation as well as attraction and tissue penetration of various leukocyte subpopulations [6]. This, in principle, enables and promotes tissue repair. Upon excessive stimulation, however, platelet-induced attraction and activation of leukocytes can aggravate underlying tissue injury in conjunction with evolving microthrombosis formation, thereby promoting perpetuating autodestructive cascades.

Whether the increased platelet count in conjunction with leukocytosis, sustained norepinephrine-mediated platelet activation, and increased retention of platelets within the brain (positive arterio-jugular venous platelet difference) contributed to the signs of cerebral deterioration as reflected by elevated ICP, decreased SjvO_2_, and sustained lactate release during the second week remains unclear.

Based on findings obtained in other neurodegenerative diseases, activated platelets could be of increasing pathophysiologic importance also following clinical TBI. As reported by Mathew and colleagues [41], transcerebral activation of platelets occurred following the release of aortic crossclamp in patients subjected to cardiac surgery and was associated with neurocognitive worsening. Altered platelet function resulting in impaired uptake and sustained release of glutamate might also promote cerebral injury as discussed for cerebral ischema [42], migraine [43], and epilepsy [44].

The finding of norepinephrine-mediated increased platelet activation during the second week with a significantly attenuated effect during the first week does not automatically imply functional disturbance of platelets resulting in additional hemorrhage or contusion growth. Further analysis, however, is required to determine norepinephrine-induced release of platelet-derived toxic mediators despite nearly unchanged expression of P-selectin in the early phase following sTBI.

## Conclusion

The present results clearly demonstrate that *in vitro *stimulation of isolated platelets is required to unmask functional alterations that are missed when considering only P-selectin and microparticle expression of non-stimulated platelets. At present, it remains unclear whether the observed alterations are of clinical importance since only norepinephrine in high concentrations exceeding clinically relevant plasma levels (>25 nM) increased the expression of surface P-selectin and intracellular microparticles in isolated platelets. The differentiated temporal profile of altered platelet activation could result from functional downregulation of α_2 _receptors during the first week followed by upregulation of α_2 _receptors during the second week, possibly explaining the preceding depressed and subsequent sustained stimulatory effect of *in vitro *norepinephrine on isolated platelets, respectively. Coinciding with the increased norepinephrine-mediated stimulation of isolated platelets, platelets appeared to adhere to cerebral endothelial cells during the second week as reflected by the positive AJVD in platelets. In addition, signs of cerebral worsening were encountered. Whether these findings are merely coincidental or indeed are of pathophysiologic and therapeutic importance needs to be investigated. It also remains to be determined whether norepinephrine should be avoided or limited to a certain dose during the second week to prevent norepinephrine-mediated platelet activation with its subsequent potentially adverse tissue-damaging effects. Future research should also investigate the pharmacodynamic profile of, for example, phenylephrine and the effects of additional administration of specific α_2 _adrenergic inhibitors such as, for example, yohimbine.

## Key messages

• *In vitro *stimulation of isolated platelets unmasks functional changes.

• Norepinephrine, in a concentration-dependent manner, stimulates isolated platelets in healthy volunteers and critically ill patients with severe traumatic brain injury.

• Stimulation was similar in arterial and jugular venous platelets.

• Isolated platelets express a temporally heterogeneous susceptibility to norepinephrine-mediated stimulation, reflected by a decreased response during the first week followed by an increased stimulation in the second week.

• In the second week, increased platelet susceptibility to norepinephrine-mediated stimulation coincided with signs of cerebral worsening.

## Abbreviations

AJVD = arterio-jugular venous difference; CPP = cerebral perfusion pressure; ELISA = enzyme-linked immunosorbent assay; HES = hydroxyethyl starch; ICP = intracranial pressure; ICU = intensive care unit; IL = interleukin; PRP = platelet-rich plasma; SjvO_2 _= jugular venous oxygen saturation; sTBI = severe traumatic brain injury; TBI = traumatic brain injury; TRAP = thrombin receptor-activating peptide.

## Competing interests

The authors declare that they have no competing interests.

## Authors' contributions

CT isolated the platelets, performed the *in vitro *analysis, and drafted the manuscript. LMA helped to analyze and interpret the data and drafted parts of the manuscript. PML analyzed the sP-selectin data. MT helped to collect data from healthy volunteers. LH provided valuable input in the ELISA measurements. MK helped to analyze the data and drafted parts of the manuscript. RS contributed to discussions of the data and drafted parts of the manuscript. JFS conceived the study design, collected parts of the data, performed graphical and statistical analysis, and drafted parts of the manuscript. All authors read and approved the final manuscript.
